# Supplementary material for: MxB sensitivity of HIV-1 is determined by a highly variable and dynamic capsid surface
Source: eLife. 2020 Jun 17;9:e56910. doi: 10.7554/eLife.56910 (PMC7299335; doi:10.7554/eLife.56910)
Supplement: Supplementary file 1. [file elife-56910-supp1.pptx]

## Slide 1
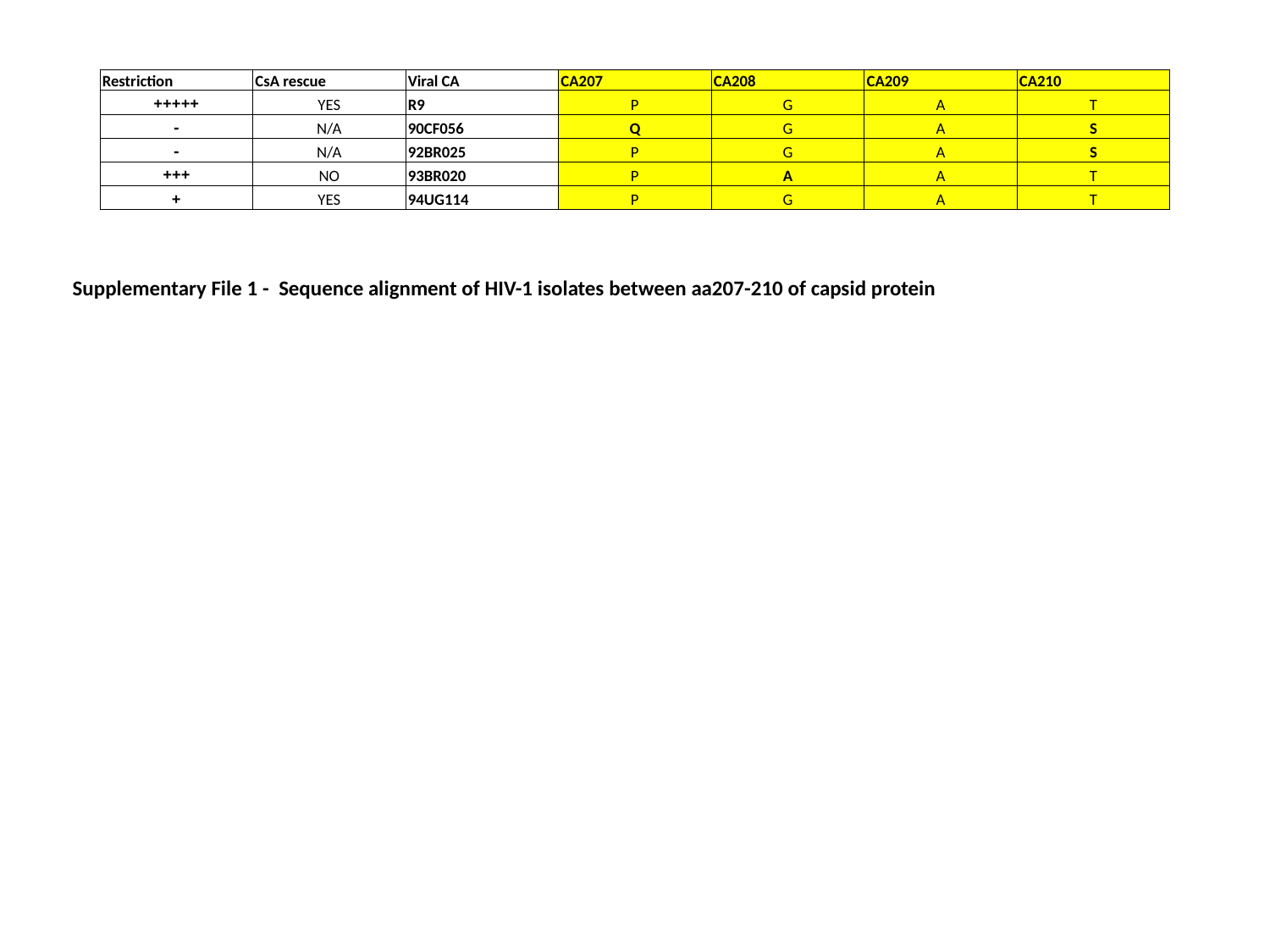

| Restriction | CsA rescue | Viral CA | CA207 | CA208 | CA209 | CA210 |
| --- | --- | --- | --- | --- | --- | --- |
| +++++ | YES | R9 | P | G | A | T |
| - | N/A | 90CF056 | Q | G | A | S |
| - | N/A | 92BR025 | P | G | A | S |
| +++ | NO | 93BR020 | P | A | A | T |
| + | YES | 94UG114 | P | G | A | T |
| | | | | | | |
Supplementary File 1 - Sequence alignment of HIV-1 isolates between aa207-210 of capsid protein
